# Early evidence for capacity standardisation in Western Europe. The vessels from Mailhac (Aude, France) 9th-7th centuries BC

**DOI:** 10.1371/journal.pone.0326354

**Published:** 2025-08-13

**Authors:** Thibaud Poigt, Alexis Gorgues, Antoine Dumas

**Affiliations:** 1 UMR 5607 Ausonius, University Bordeaux Montaigne, Pessac, France; 2 UMR 5607 Ausonius, Inrap NAOM, Pessac, France; Austrian Academy of Sciences, AUSTRIA

## Abstract

This paper presents an original study of the metrological characteristics of a series of vessels discovered in the necropolis of *Le Moulin* (Mailhac, southern France) and dated to the Late Bronze Age and the beginning of the Early Iron Age. A metrological study of the capacities of these artefacts is presented, based on a protocol of 3D modelling from 2D drawings to calculate the internal volumes of the vessels, and a series of mathematical and statistical analyses. The results make it possible to identify one of the earliest evidence for metrological practices based on capacity in Western Europe.

## 1. Introduction

The interest in ancient metrology in Western Europe is as old as the first observations on Bronze and Iron Age artefacts [1–3]. Since then, our knowledge of Late Prehistoric measures has increased considerably, especially due to the progressive construction of large collections and the development of new methodological approaches [4–8]. Nevertheless, the majority of this research has only been related to the metrology of weighing, namely through the study of weights, balances and metallic artefacts that are metrologically standardised.

The number of studies devoted to length and volume measurement is surprisingly small in comparison [9–16]. Consequently, we generally consider quantification practices only from the perspective of weighing, neglecting the use of alternative quantification methods. For example, considering Western Europe, the use of vessels with standardised capacities is generally attributed to the Phoenicians or Romans and their trading activities. The hypothesis that non-literate peoples developed plural metrologies (based on several physical characteristics) is rarely proposed.

Nevertheless, ethnological and historical works show that there are probably no ‘non-measuring’ societies [17]. Indeed, there is a wide variety of aspects of daily life that involve concepts of measurement, from the act of quantifying the time, amount of seeds and area of field needed to feed a human group, to calculating the benefits of organising a maritime expedition.

**Fig 1:**
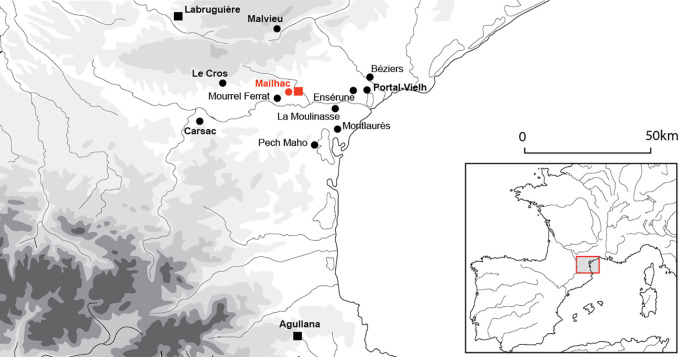
Localisation of Mailhac and the neighbouring settlements of the Bronze Age/Iron Age transition. CAD and background map: A Gorgues.

This paper presents a case-study from Southern France that shows evidence for capacity metrology in contexts dating back as far as the Late Bronze Age and the Earliest Iron Age (10^th^-8^th^ centuries BCE). It is based in particular on the study of vessels’ capacities from the necropolis of Le Moulin at Mailhac (Aude, France) (Fig 1).

## 2. Mailhac (Aude, France) in context: 950−700 BCE

**Fig 2:**
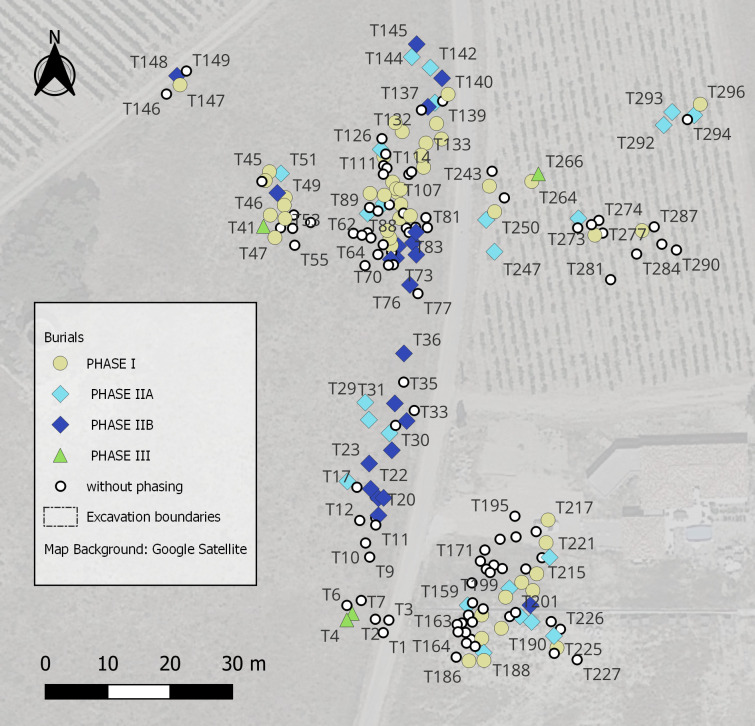
Map of *Le Moulin* burials depending on their chronological phasing. GIS: A. Dumas, T. Poigt.

Malihac archaeological complex comprises a hilltop settlement (Le Cayla, occupied from *c.* 950 to the Roman period, with a hiatus between *c.* 750 BCE and *c.* 575 BCE), a lowland settlement (Le Traversant, mainly occupied from the end of the LBA to *c.* 575 BCE) and a necropolis divided into three *nuclei* each corresponding to a specific phase (Le Moulin, *c.* 950−700 BCE, with some tombs being slightly later; le Grand Bassin 1, *c.* 700−575; le Grand Bassin 2: *c.* 575−450 BCE: last synthesis in Beylier *et al.* 2024 [18]). This paper focuses on the Late Bronze Age and Early Iron Age vessels found in the cemeteries of Le Moulin, where some 370 graves (Fig 2) out of a maximum of 1000 were excavated between 1948 and 1973 [19].

From the end of the Bronze Age to the very beginning of the Iron Age, the period addressed in this paper, the Mailhac complex was part of a wider ensemble which, overall, extended eastwards from the Orb basin (in what is now the Hérault *département*) westwards to the Toulouse and Albi regions, and southwards to the Empordá (Catalonia, Spain). More than a homogeneous horizon, it was a cluster of micro-regions interconnected by similarities in funerary practices [20] and techniques, particularly pottery [21,22]. In this ensemble, large necropolises appeared, especially from the 10^th^ century BCE onwards, which would contain hundreds of cremation tombs accumulating over several centuries. Settlement patterns were structured, at least in part, around hilltop sites, often fortified [23]. Beyond these general trends, micro-regional nuances could be strong, at least in the current state of our knowledge. For example, large necropolises rarely flank habitats of proportionate size (it only happens in Mailhac), and the reverse is also true: no large necropolis is known to be associated with the important site at Carsac, and no important dwelling is known near the large necropolis at Castres. Around Malvieu, which was occupied from at least the 11^th^ century BCE, there are only two known graves dating from the very end of the site’s occupation, around 500 BCE, and even then at some distance. Some hilltop sites seem to have been deserted by the end of the Bronze Age, around 800 BCE (Le Cayla, for example), while others continue to be occupied (Malvieu for sure, perhaps Carsac). The size of the sites can vary quite considerably, from 1 or 2 ha (Malvieu) to around 20 ha (Carsac). Le Cayla occupied surface is supposed to measure between 4 and 6 ha. Inner occupation could be dense (Malvieu) or looser (Carsac); it is poorly characterised for these periods at Cayla. In this varied landscape, the elements of continuity between the end of the Bronze Age and the beginning of the Iron Age are more significant than those of rupture [23], with a general trend, between the 10^th^ and 6^th^ centuries, towards the accentuation of social differentiation which can be seen both in the funerary record and in that of the habitat. The period between the end of the Bronze Age and the beginning of the Iron Age therefore corresponds, in our study area, to the initial stages of the formation of the societies that would later become those of the full Iron Age (6^th^-3^rd^ centuries BCE).

Mailhac archaeological complex is quite emblematic of this. Despite the abandonment of le Cayla around 800 BCE, the occupation continued until the 6^th^ century BCE with the development of a lowland settlement centered on Le Traversant, and funerary activity never ceased until the end of the 1^st^ Iron Age [18]; this is one of the rare cases where a settlement and a proportionate necropolis developed in parallel over a small area. In Le Moulin, first signs of differentiation can be seen, but this process will accelerate in the later funerary complexes of Grand-Bassin 1 and 2.

The case study we are proposing here therefore focuses on the first centuries of a community whose history will extend into Roman times. Contrary to what we might expect in other regions of the western Mediterranean, these initial dynamics owe little to maritime contacts. Although the societies of the late Bronze Age and the very early Iron Age obviously practised trade, contacts with other regions of the Mediterranean still seem very limited [24]. It was not until the 2^nd^ half of the 7^th^ century BCE that the connectivity of this region increased significantly, as demonstrated by the so-called ‘Launacian’ deposits and some exceptional finds, as the Rochelongue shipwreck [25,26]. The mobility of people seems to have played a major role in this process, which led to the connection of regions that were very far apart [23,27,28]. The impact of these contacts on the ceramic repertoire remained limited until the 6^th^ century BCE, and was confined to the occasional imitation of forms from the western Phoenician domain [29].

## 3. Methodology

The protocol used in this study is divided in two successive steps: data collection and data analysis. The data used here are based on published material: drawings of vessels and their measurements. The main challenge is to calculate the internal volume of the vessels from 2D drawings.

**Fig 3:**
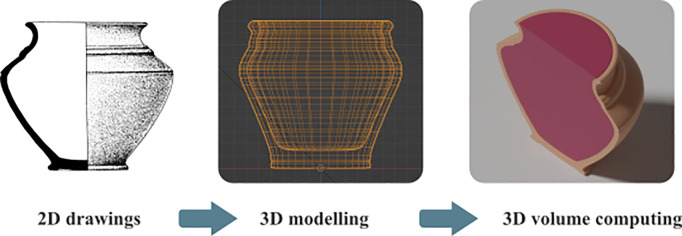
Schema of the 3D modelling protocol from 2D drawings. Based on the add-on profil2volume written by F. Comte, with upgrades from A. Dorkel and T. Poigt.

To achieve this, we rely on a 3D modelling protocol (Fig 3) developed in the framework of the NoStOi project (Norms, Standards and Routines: pottery production and information in the Ancient Mediterranean), coordinated by one of us, Alexis Gorgues, between 2016 and 2021 [23,30]. The process consists of copying the section of the vessel within the software Blender by importing a scaled drawing extracted from the necropolis publication and reproducing it as a series of connected vertices on two dimensions. This section is then rotated through 360° to reproduce the complete vessel including its shape and its content (up to the top) as two distinct closed meshes. From them, it is possible to extrapolate the vessel capacity to the top as well as the volume of clay used to manufacture it [31]. It is computed on the free software Blender with the help of an add-on that automates the tasks, developed by Florent Comte (UMR MAP, ERC Advanced Grant n-Dame_Heritage) and improved to the latest versions by Alexia Dorkel (University of Geneva).

Other techniques exist for calculating vessel capacity, without extrapolating it from drawings. These include filling vessels with a semiliquid material and scanning them directly using photogrammetry or a structured-light 3D scanner. Nevertheless, none of these techniques is adequately suited to the analysis of the Mailhac corpus. In both instances, this methodology can only be applied to complete vessels; whereas, the 3D modelling through 2D drawing only necessitates a complete section. In the case of vessels that are nearly complete but fragmented, the precision of the calculation is contingent upon the quality of the reconstruction, which is typically oriented towards museum rather than scientific objectives. Similarly, digital restorations are required when 3D scanning vessels that are incomplete in order to calculate their capacity.

In consideration of the sample from Mailhac, it can be observed that the vessels are, in general, fragmented and frequently incomplete in some aspects. The most complete vessels have been restored through the application of an adhesive to the sherds and the introduction of resin-based material to fill the gaps. If the objective of this technique is to present a complete exterior aspect in museum exhibitions, it also results in significant modifications to the inner capacity, with the application of thick layers of resin. In several cases, it fills entirely the bottom of the vessel, altering drastically its capacity. A comparative test was conducted between models obtained through photogrammetry and those created using our own protocol. However, the inconclusive nature of this test was due to the current state of the vessels as well as technical difficulties to model the inside of the vessels.

In comparison, our protocol presents two main advantages. Firstly, it is based on scientific documentation, which is rigorous, published, and therefore easily available and reusable. Secondly, it allows for the processing of a large sample in a relatively short period of time (around 50–60 vessels per day), with the results exported directly as a series of volumes that can be subjected to metrological analysis.

Hypothetical metrologies are thereafter inferred and tested by two main analyses: the Frequency Distribution Analysis and the Cosine Quantogram Analysis. These steps have been conducted with the “Metrological ToolBox”, developed by one of us [32] in the framework of a PhD thesis [33] and regularly improved since.

Frequency Distribution Analysis (FDA) aims to visualise clusters of values: a sample with a metrological distribution is generally characterised by a high number of values around a standard, its fractions and its multiples. To take account of the various errors that can accumulate (precision of the original unit, manufacture of the artefacts, conservation, drawings and modern measurements), we observe these values with a proportional tolerance, generally set at 5%. For example, the number of values for 10l actually shows the number of vessels with a volume between 10l minus 5% (9.5l) and 10l plus 5% (10.5l). This graphical visualisation is more suitable for a sample composed of several arithmetically interdependent values, which can accumulate several errors and tolerances. To observe the main clusters, taking into account a specific metrological system, several tolerances are cumulated on the same graph, from 1 to 10%.

The Cosine Quantogram Analysis (CQA), developed by David G. Kendall for archaeological studies, is a mathematical and statistical method based on a “Fourier transform” that allows to test if a single value, called *quantum*, can be considered as a structuring unit for the largest number of data within a sample. Several values are tested in this way, allowing proposing several *quanta* for the sample [5,34,35], based on the following formula:


$$\phi (q)=\sqrt{\frac{2}{N}}\sum_{i=1}^{n}\cos\left(\frac{2\pi \varepsilon_{i}}{q}\right)$$

In more detail, CQA tests whether an observed measurement *X* is an integer multiple of a *quantum q* considering an error *ε*. For each value of *q*, a value *Φ* is calculated. Positive results indicate a low error *ε* and *X* is close to an integer multiple of the *quantum q*. In other words, the higher *Φ* is for a value *q*, the more likely it is that this value structures the sample as a metrological unit.

The significance of the results can then be defined through a Monte Carlo simulation. It is managed by the generation of 100 randomized datasets with 0.15 deviation of the original archaeological sample. Each one of these is subjected to a CQA, recording its Phi value. The significance threshold is determined by the number of simulations providing a Phi value higher than the original one.

The CQA analysis can produce false positive results, especially when applied to small samples. Nevertheless, we rely on a protocol going back and forth through CQA and FDA to control the reliability of the results. This approach has been specifically developed to deal with small samples [33], and more precisely, smallest samples that the ones tested in this paper. The previous studies using this mix of analysis provided very good results, avoiding to give too much weight to the CQA alone.

## 4. Corpus

### 4.1. Ceramic vessels characteristics

**Fig 4:**
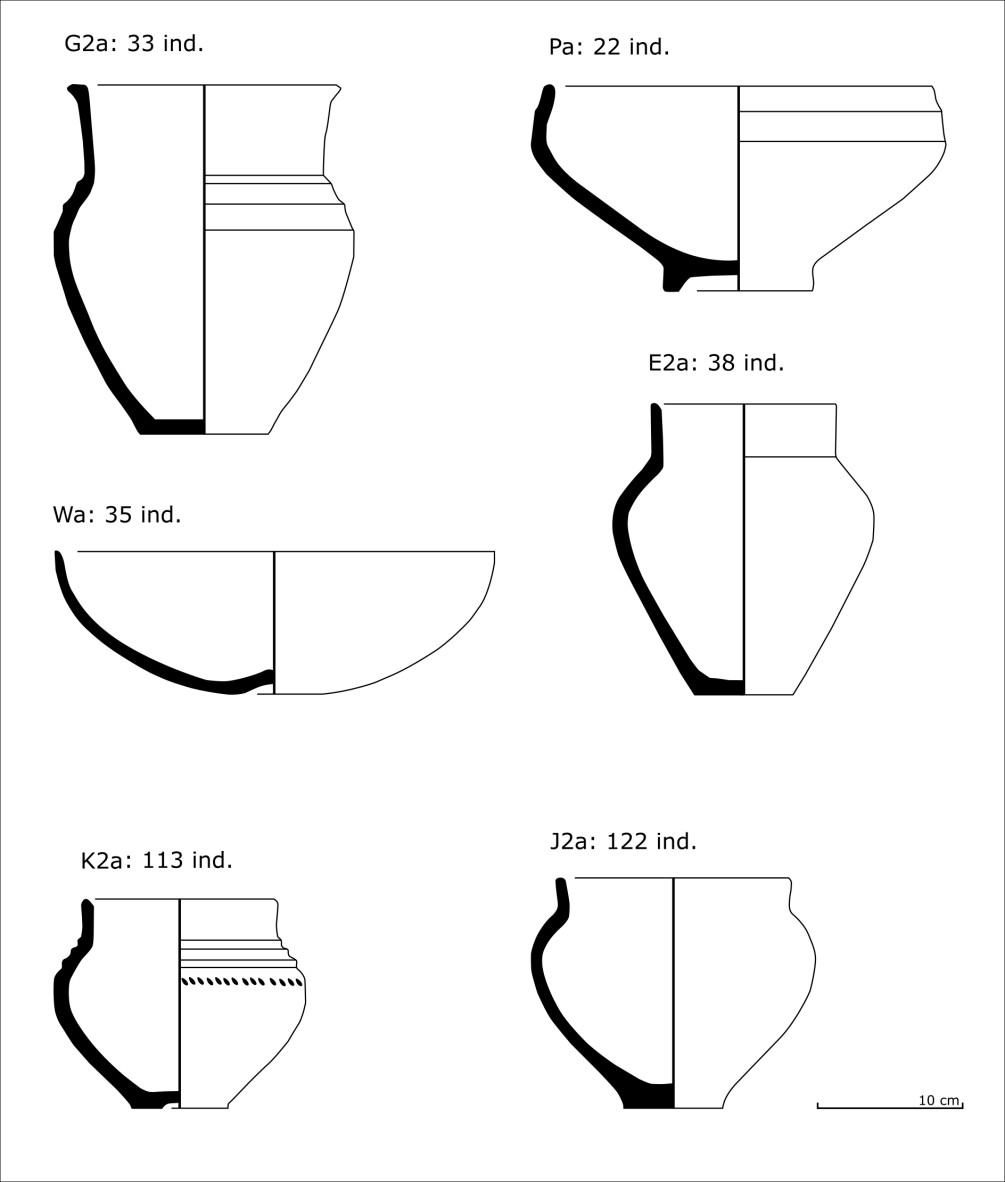
Examples of the main categories of vessels provided by *Le Moulin* burials studied in the paper. Drawings: Taffanel *et al*. 1998; CAD: T. Poigt.

The necropolis of Le Moulin provided 620 vessels from 224 different burials (S1 Appendix), an entire section of which can be modelled in 3D. The 2D drawings used as a basis for the 3D reconstruction were taken from Taffanel *et al.* 1998 [19] (Fig 4). The sample is spread throughout the activity of the necropolis, with some phases better represented than others. Most of the tombs cannot be assigned to a specific phase (121 tombs), one out of five belong to Phase I (900−775 BCE: 46 tombs), while the others can be dated to Phase IIA (775−750 BCE: 26 tombs), Phase IIB (750−725 BCE: 24 tombs) and Phase III (725−650 BCE: 7 tombs) [36,37]. This proportion does not change significantly when considering the number of vessels: 40% are undated (246 ind.), 24% are dated to Phase I (149 ind.), 15% and 16% to Phases IIA and IIB (96 and 100 ind.) and 5% to Phase III (30 ind.).

In the original publication [19], the typological analysis of the ceramic vessels was based on a method developed by A. Nickels [38] and designed for the study of the vessels from the nearby cemetery of Le Peyrou in Agde. Nickels’ method has since been used to analyse several ceramic corpuses from the burial sites of the Languedoc region, as the necropolis of Camp d’Alba (Réalville, Tarn-et-Garonne) or the necropolises of Castres (Tarn) [39,40]. In this paper, we kept the typology produced by the original authors, even if small critics can be made.

The original protocol of typological classification is quite simple and relies on a combination of a few features based on metric ratios or empirical descriptions. First, the vessels corpus is divided into two main groups: the open shapes and closed shapes. This distinction is based on an openness index equal to: total height/ maximum rim diameter. Shapes are considered closed if this ratio is greater than 0.6, otherwise they are considered open.

The next steps combine the description of the body, the neck and the base of the vessel. The different body shapes are grouped into families named with letters from A to Z. They correspond to elementary shapes, defined empirically. For example, the family D includes the vessels with a tronconic body with convex profile, while the family F is composed by bitronconic bodies with high carination. Unsystematically, some metric ratios may play a part in the definition of types. For example, the families E and H are vessels with ovoid bodies, but are distinguished by the relative position of the maximal diameter, expressed as the ratio body height/ maximum diameter height.

Subgroups are then defined within each family on the basis of neck size. The neck height index is defined by the ratio neck height/ diameter of the neck base. Three types are defined: small necks (1: index < 0.08), medium necks (2: 0.08 ≤ index ≤ 0.5), large necks (3: index > 0.5).

The last step of the protocol takes into account the base of the vessel, through the index expressed by the ratio: height of the base/ diameter of the base. Three categories are defined: undeveloped bases (a), low supports (b: index < 0.26) and high supports (c: index > 0.26).

The morphological types are named by the concatenation of the three features (D1a, E2b, J1a, etc.) corresponding to the three descriptive steps of the shape: family (A to Z) + collar (1–3) + base (a to c). Considering the necropolis of Le Moulin, the application of this method led to the definition of 50 different morphological types. All the elements of decoration are subject to an additional description for each type.

This method has several disadvantages and limitations. Firstly, it requires archaeologically complete sections, therefore incomplete forms cannot be properly described. In the case of Mailhac corpus, this issue is anecdotal as most of the vessels are archaeologically complete.

Secondly, it ignores the morphology of the rim, whilst it can provide chronological information. Again, this is not consequential for Mailhac, as the data are complete enough to allow a seriation of the corpus.

Thirdly, the definition of the families as elementary shapes, characterised empirically, conducts to a great variety within each family. It sometimes leads to unexpected groupings or distinctions between two shapes that could be considered close to each other. Such a variability must be considered as a normal consequence of the corpus being composed by handmade pottery. Taking this criterion into account, the formal coherence of the different types of the whole corpus remains quite satisfactory. Furthermore, the choices made by the authors for the attribution of the families rely on a deep knowledge of the corpus from Mailhac as well as other reference assemblages from south-western France. The results of the typo-chronological analysis presented in the Mailhac monograph [19] confirm the validity of the method chosen, even if it could be slightly modified. Therefore, the data resulting from the typological classification are used without modification in our study.

We know little, if any, about where LBA pottery was made (on the contrary, for the EIA around Mailhac [41]). It was hand-made pottery, with strong local peculiarities (among which a specific decorative style), but belonging to a much wider complex encompassing most of southern France [21]. Influence of Mediterranean connectivity over the morphological repertoire is nil before 650 BCE, and quite limited before the potter’s wheel was introduced in the region during the 6^th^ century BCE.

### 4.2. Volumetric features of the corpus

A number of error factors can affect the calculation of the internal volume of a vase. In order to enhance the precision and validity of our analysis and results, we sought to identify these errors and their impact on the data, with a view to producing cleaned and reliable samples. In particular, we list a series of metrics that could be compared between the data published and the 3D models obtained through modelling.

First, a series of errors or potential biases can be detected within the published documents. To begin with, even when the author has drawn a complete section of the vessels, some of them are missing parts at the top, foot or body, leading to probable distortions. Additionally, several errors concern the scale of the drawing or general metrics. Anomalous measurements of their height, opening diameter or foot diameter may be due to typographical errors, especially when the relationship between height and diameter cannot be related to the drawing in any way. In the case of several vessels, some of the required morphometric data are absent, thus preventing the verification of consistency between the 3D models and the published data.

Some vessels can also be considered too small to ensure the accuracy of their measurement on a 3D basis. The authors of Mailhac monography measured heights and diameters to an accuracy of 5 mm. We can therefore conclude that they rounded up to 2.5 mm at best. The “resolution” is therefore lower for the smallest vessels than for the largest. For example, it implies a 10% approximation for a measurement of 2.5 cm. For this reason, and to avoid the distortions which the smallest artefact could bring, we exclude the vessels with heights or diameters of 3 cm or less. According to these criteria, the sample is reduced to 428 vessels.

Such a withdrawal does not present a significant obstacle to the analysis, particularly given the observations of Ialongo and Lago [42], who demonstrated that the introduction of data below the metrological unit threshold could result in false positives with regard to the CQA. Nevertheless, these data may be reintroduced in a subsequent step dedicated to the metrological interpretation of the sample structure.

There are also errors that cannot be detected from the published drawings and their descriptions. The original drawings may also contain errors and approximations. The 3D modelling protocol itself is biased because it involves scaling, re-drawing of the section and smoothing the mesh and volume. More importantly, our process is based on a 360° rotation of a section, which assumes that the vessel is perfectly symmetrical. However, the vessels from Mailhac are not made with a wheel: their symmetry is not guaranteed, and sometimes was clearly not achieved. Finally, human approximation or error is likely to occur at every stage of the process, deviating the real capacity of the artefact from its ideal one. It is true for the making of the vessel, but also for the study protocol.

All these points emphasise the importance of calculating the deviation that may exist between the data gathered and the real capacities of the vessels. To do this, we compare morphometric data given in the publication with measurements taken directly on the 3D models.

For the 620 vases modelled, 1752 measurements (height, opening diameter, base or foot diameter) are given by Taffanel *et al.* 1998 [19]. They can be compared with 1731 measurements made on the 3D models (bases are sometimes hardly measurable on the 3D meshes and have been discarded when uncertain). 1605 values are common to both processes. Average absolute deviation between these values is 4.51%, very acceptable for ancient metrology studies. If analysis is limited to the 428 vessels of the cleaned corpus, 1264 published measurements can be compared with 1224 3D measurements, providing 1140 common values. The error drops to 2.75%.

Furthermore, it is worth emphasising that scaling is mostly based on the height of the vessels, which logically deviates less from the published data. Deviation calculations taking only into account the opening diameters (measurement of the base are a lot less accurate) return an absolute average deviation of 2.95%, based on 423 common measurements between 426 published measurements and 428 3D measurements.

**Fig 5:**
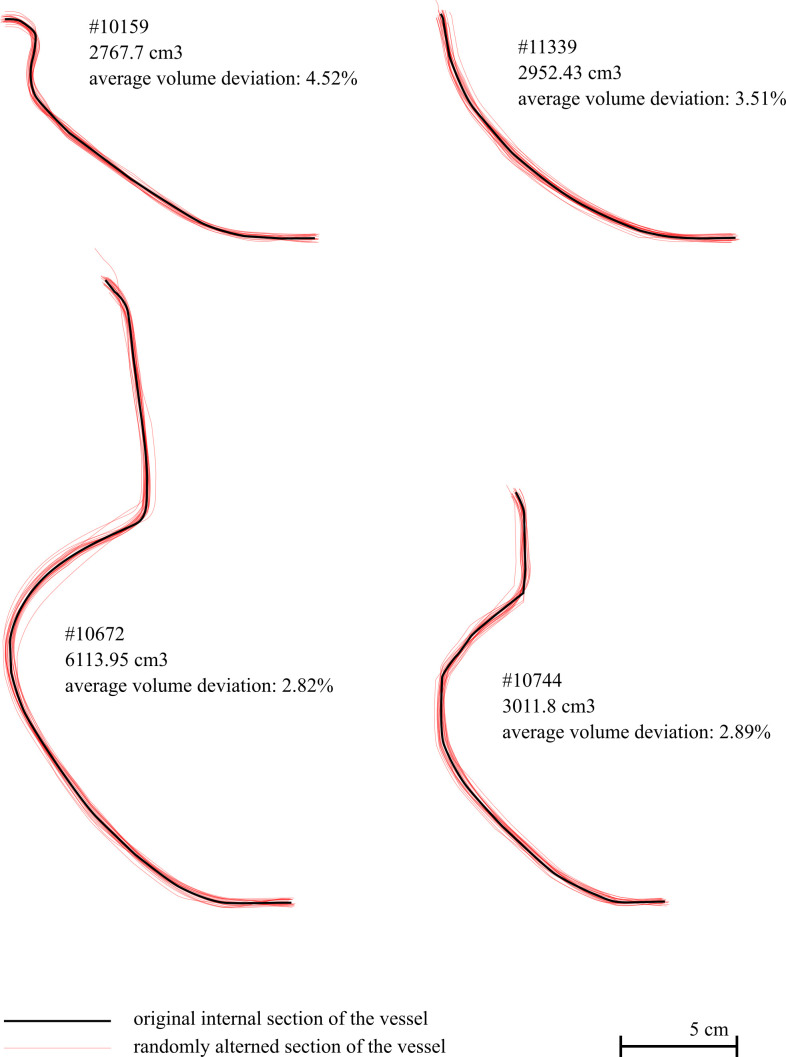
Illustration of the asymmetry tests. Carried out on vessels #10159, #10672, #10744 and #11339 with the superimposition of their original internal sections (in black) and 30 simulations of altered sections (in red).

A last question concerns the impact of asymmetry on the calculation of capacity through a 360° spin of one section. To evaluate such an impact, we conducted a test with 4 vessels of different types. For each one, we produce a series of 30 altered copies of their internal section (Fig 5) that simulate deviation from the “normal” section that can occur with handmade asymmetric vases, with a consistent order of magnitude. For each one, 10 of the clones have been manually modified, by moving vertices, whilst the 20 others have been randomly modified through a script by automatically moving the vertices. All the sections obtained have been subsequently modelled to calculate their capacity in the same way than the original ones. The relative deviations between the altered vessels and the original ones range between 0.13% and 11.58% (S2 Appendix). The average deviation for each vessel are quite low: 2.82% and 2.89% for the closed shapes and 3.51% and 4.52% for open shapes. These values correspond to the deviation of two symmetrical vessels, one with and the other without alteration. In the case of an asymmetrical vessel, we consider that one section is unaltered and the opposite section is altered, meaning that the numbers need to be divided by two, i.e., a range of deviation between 0.07% and 5.79%, and average deviations of 1.41% and 1.45% for the closed shapes, and 1.75% and 2.26% for the open shapes.

These numbers are low enough to consider that our protocol, even if it ignores the asymmetry, can be applied to handmade asymmetrical vases without strong distortions.

All in all, the deviation induced by our protocol is perfectly acceptable for ancient metrology studies. In fact, it is comparable to what would be expected from a potter anticipating the internal capacity of a vessel during its manufacture and before firing. Today, most commercial regulations allow a 5% deviation from the stated quantities, while experimental work on Roman wheel-thrown pottery has shown that a 3% deviation can be achieved with minimal tools [43].

Nevertheless, a more refined sample will be used to compute the CQA in order to optimise the clarity and intelligibility of the results. In order to achieve this, we exclude from the clean sample the vessels with an opening diameter that differs from the published data by 6% or more. Indeed, the diameter of a vessel is the parameter for which the slightest variation has the greatest impact on the inner capacity. Consequently, the opening diameter represents the most suitable data set for the refinement of the sample. The resulting sample comprises 387 vessels.

This study represents a first attempt to explore the markers of the earliest capacity measurements and the way in which the validity of the data can be certified. In future research, we can expect to obtain a better estimate of the deviation between the calculation of the internal volume based on the 2D drawing of the original artefacts and the real capacity. During the preparation of this paper, a first attempt was made using photogrammetry. However, the sample obtained was too small and inaccurate to be used. To go further, it will be necessary to accurately digitise the vessels studied, but the corpus from Mailhac is not the most suitable for this task.

## 5. Metrological analysis

Unless otherwise stated, the FDA are based on the broad “cleaned” sample, which consists of the internal volumes of 428 vessels, and the CQA are based on the refined “cleaned” sample composed by 387 vessels.

### 5.1. General features of the distribution

The initial stage of the process is to evaluate whether the sample of capacities exhibits the characteristics anticipated for a model that is based on metrological considerations.

**Fig 6:**
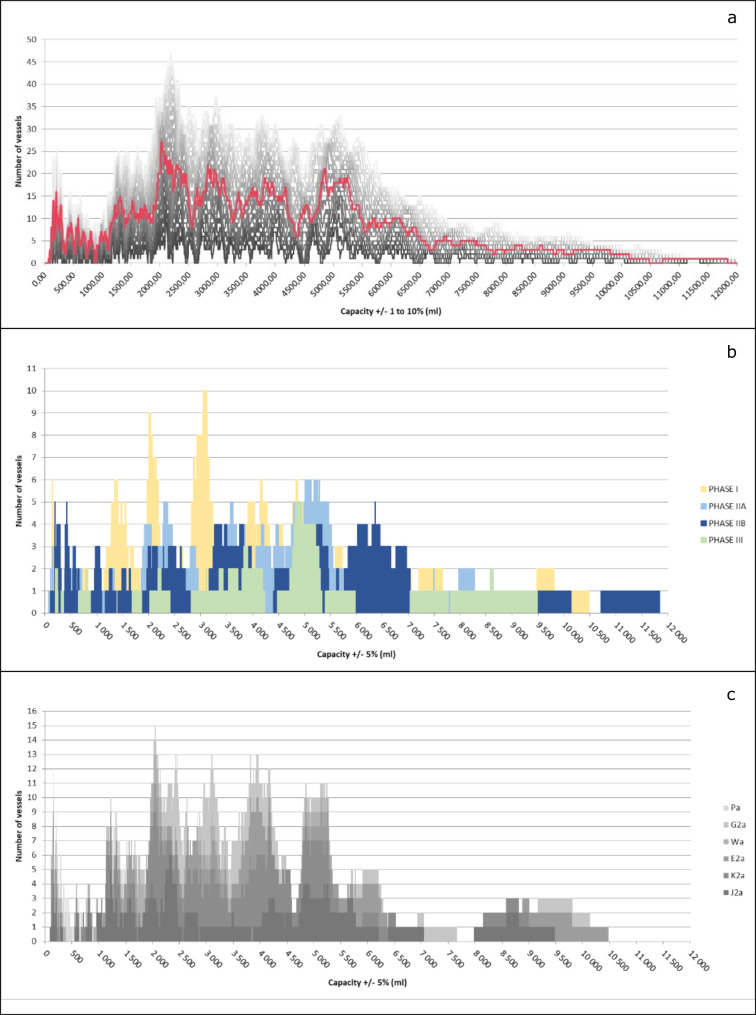
Frequency Distribution Analyses. (a) Frequency Distribution Analysis of the entire cleaned sample (428 ind.) with tolerances from 1 to 10% (5% in red). (b) FDA (cluster chart) of the vessels assigned to a specific phase (277 ind.). (c) FDA (stacked chart) of the vessels corresponding to one of the six most represented types (231 ind.).

A first analysis shows that the sample does not conform to what we generally call a normal or Gaussian distribution. Indeed, on closer inspection, we can see that the sample is not distributed around a single value, but around several. The FDA of the corpus, with a 5% tolerance, shows a series of peaks and troughs that are typical of a metrological distribution, but can also be caused by other phenomena (Fig 6a). A Kolmogorov-Smirnov test allows us to statistically reject the hypothesis that the sample is normally distributed for α = 0.01.

By splitting the sample, we can check whether such a distribution is a consequence of the intrinsic characteristics of the data. Specifically, one wants to verify if the sample is not composed by several sets following normal distributions, but each one centred around a specific value.

**Fig 7:**
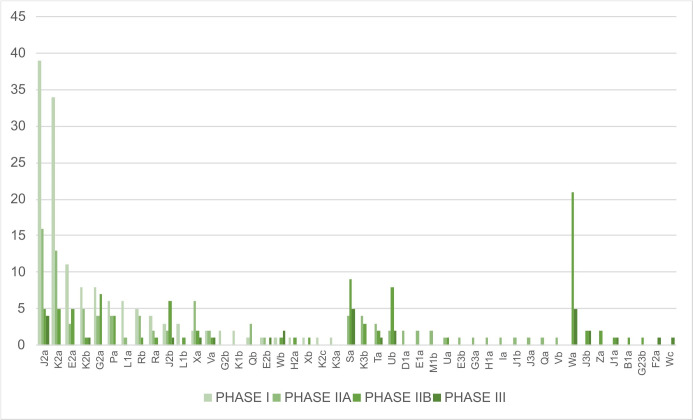
Number of vessels studied depending on their type and chronology.

In particular, since dating is partially based on pots’ typochronology [19] (Fig 7), a possible influence of the chronology on the distribution could be expected (Fig 6b and 6c).

**Fig 8:**
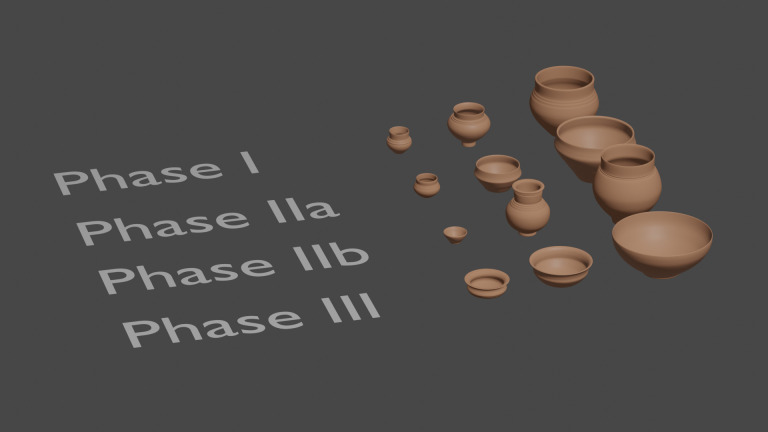
Example of vessels with different sizes and capacities for each phase. c. 100-250 ml, 450-1000 ml and 2500-3000 ml (3D modelling: T. Poigt).

**Fig 9:**
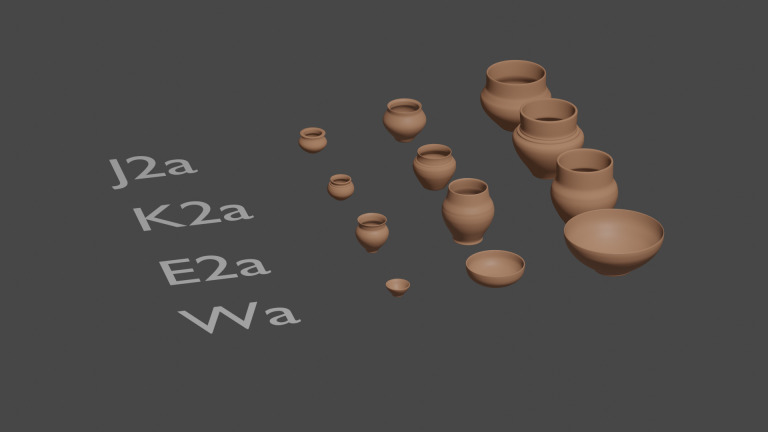
Example of vessels with different sizes and capacities for the four main types. c. 100-250 ml, 450-1000 ml and 2500-3000 ml (3D modelling: T. Poigt).

Typology is also not directly related to capacity. Among the most common types of vessels, there is no correlation between capacity and type (Fig 6c). In other words, each type of vessel seems to exist in different sizes, which mostly correspond to the same capacity values (Fig 9). This observation implies that the capacity of vessels is not directly related to their function or shape and that we cannot attribute the fluctuations of capacity to evolutions in the use of specific vessels or the appearance of new ones.

Our results therefore provide good evidence for a distribution of capacities that cannot be explained by chronological or typological features. We observe four main clusters of values around 2025 cm3, 2830 cm3, 3815 cm3 and 4850 cm3 (25, 21, 18 and 21 values respectively) and at least five other smaller peaks. However, these data do not show any intuitive arithmetic consistency.

### 5.2. In search of metrological consistency

**Fig 10:**
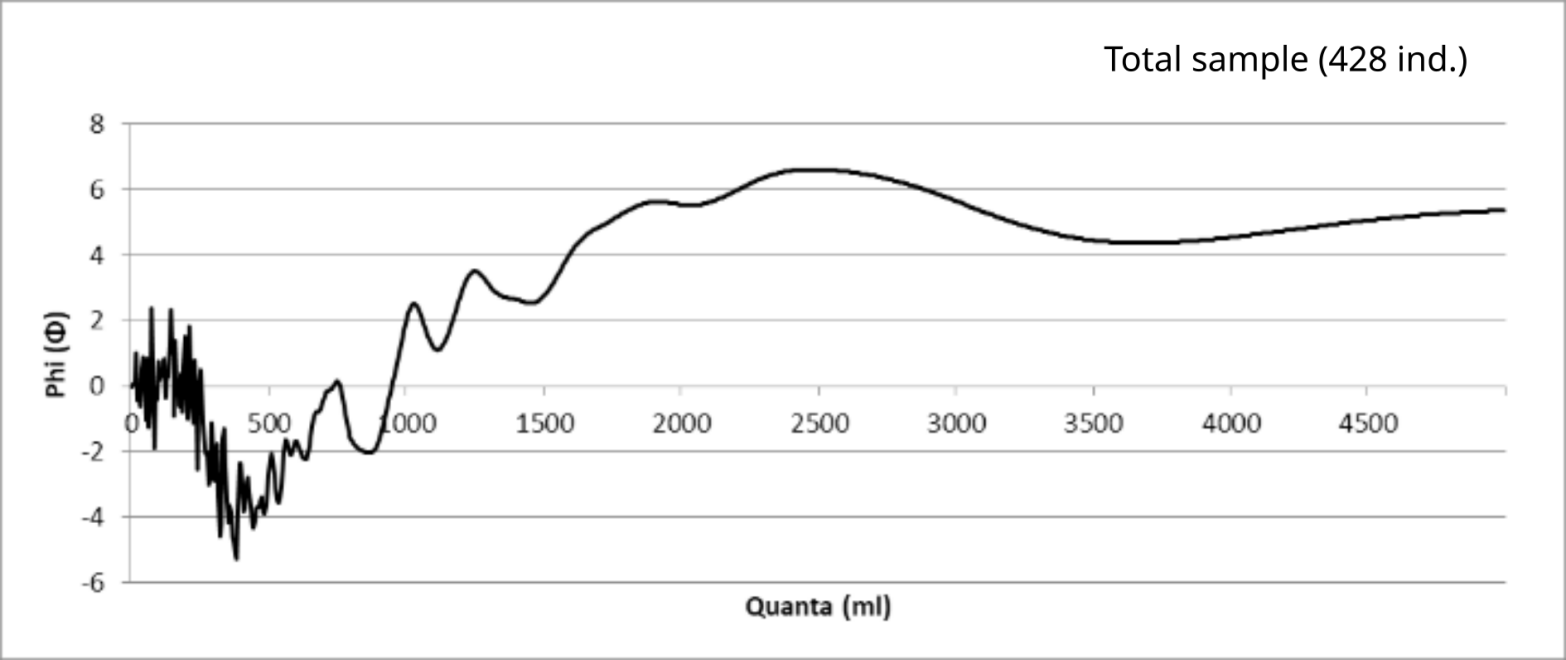
Cosine Quantogram Analysis of the entire cleaned sample (428 ind.).

As a second step, the sample is re-analysed using CQA. However, despite what the FDA could suggest, the raw analysis of the entire “cleaned” corpus is not intelligible and no guaranteed *quantum* can be highlighted from the curves (Fig 10).

These results can be interpreted in three different ways. The first possibility is that no metrology was considered by the pot makers; the clusters of values and peaks would therefore be random. A second possible explanation is that some metrology did indeed structure the sample, but according to several different units, or units that do not have intuitive arithmetical relationships. Similar situations are known from history and are not uncommon, especially for capacity measurements [44]. A third possibility is that a unit partially structures the sample, but not the whole ceramic production.

The FDA has demonstrated that the capacities of vessels are, at least partially, contingent upon the chronological phasing, which can suggest a transformation in metrological considerations over time. This is consistent with the hypothesis that multiple units structure the sample or particular phases.

Conversely, the FDA of the principal categories of vessels exhibits no profound disparities that could suggest the use of metrological units for a subset of vessels and not for others. In the preparation of this paper, an attempt has also been made to apply various spatial classifications in order to identify any potential correlation between the location of a grave and the capacity of the vessels it contains. No evidence of such phenomenon has been observed, and thus these fastidious steps are not presented in detail here.

Therefore, if a unit structures a portion of the sample, or if multiple units are used within the sample, the FDA suggests that the sole criterion for subdividing the sample from a metrological perspective is the chronology. Indeed, there is no other evidence to support the hypothesis that a unit structures only one part of the sample, whether we consider typological or spatial criteria.

**Fig 11:**
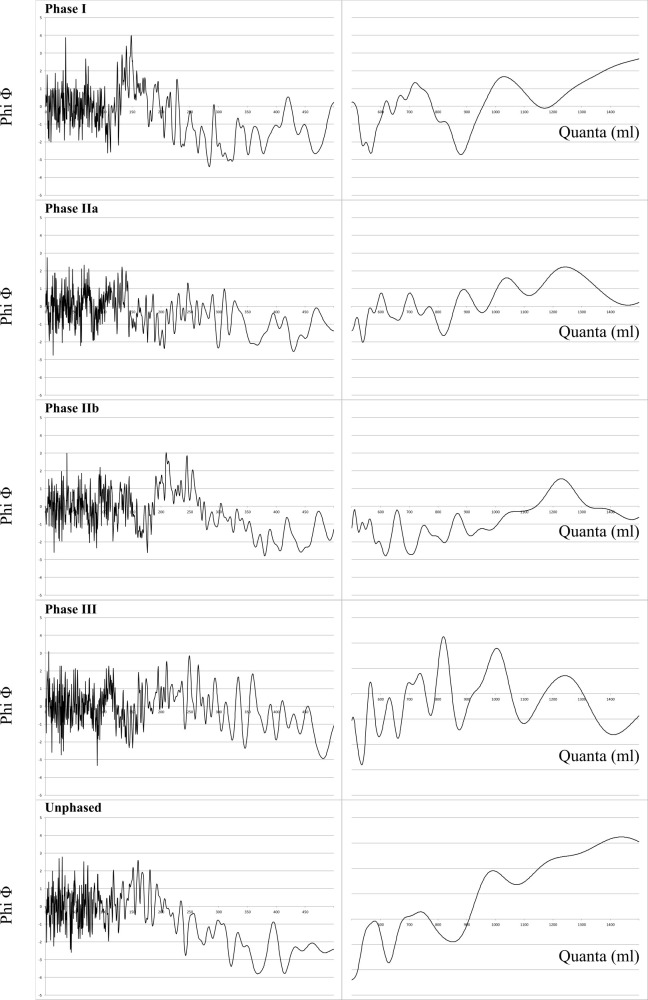
Cosine Quantogram Analysis of the sample for each phase considered independently.

In order to ascertain which of the competing hypotheses is the more probable – i.e., the absence of metrology or a metrological practice that evolves over time through the adoption, abandonment or shifting of metrological systems – the CQA is carried out once more for the different phases identified at Mailhac, independently and grouping them in a chronological order (Fig 11 and Table 1), each one observed on the intervals 0–500 ml and 500–1500 ml.

**Table 1:** Synthetic table of the CQA depending on the phases considered and their main results.

	Nbr	0-500 ml		500-1500 ml		Significance
		*Quantum* (ml)	Phi	*Quantum* (ml)	Phi	
Phase I	92	**148**	**3.83**	/	/	5% level (50–250 ml)
Phase IIa	69	/	/	1229	2.34	not significant
Phase IIb	67	245	3.07	1227	1.34	not significant
Phase III	25	249	2.96	819	2.16	not significant
unphased	134	/	/	/	/	not significant
Phase I-IIa	161	140	3.29	1037	2.02	not significant
Phase IIa-b	136	241	2.55	**1227**	**2.61**	1% level (900–1500 ml)
Phase IIb-III	92	210	3.62	1230	1,65	not significant
Phase II-III	161	246	2.69	**1229**	**2.78**	1% level (900–1500 ml)

**Fig 12:**
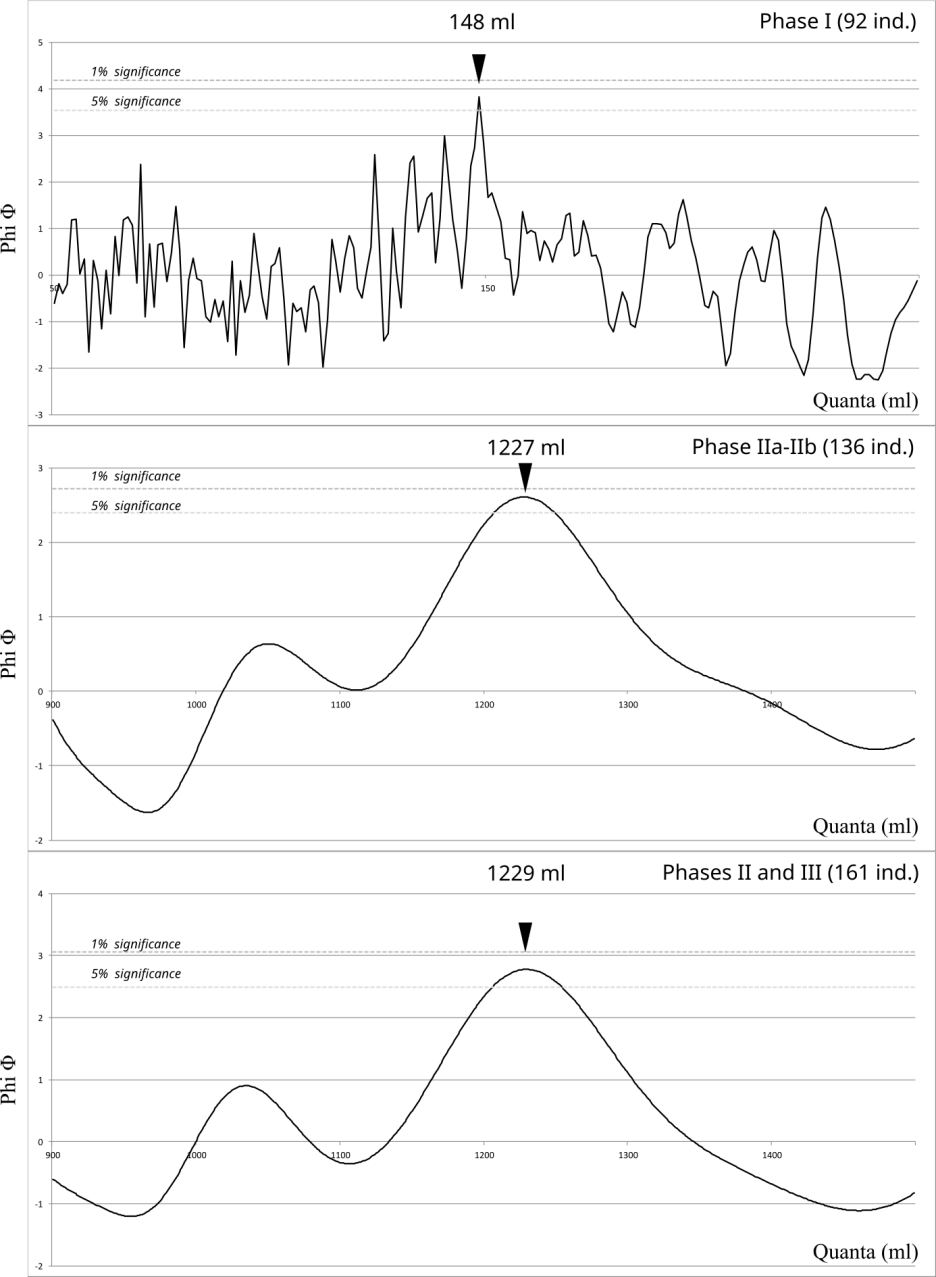
Cosine Quantogram Analysis with 1% and 5% significance levels. Vessels from Phase I (92 ind.); the vessels from Phase II (136 ind.); vessels from Phase II and III (161 ind.).

The results of these analyses suggest that some metrological evolution is observable from a chronological perspective. If each chronological group has been tested, we will only discuss the ones that suggest metrological structures and which are consistent with the phasing of the necropolis. When analysed individually, only the data from phase I yields a notable result with a *quantum* of 148 ml (within the range of 50–250 ml above the 5% level). By grouping the different phases together, two further noteworthy outcomes emerge: a *quantum* of 1227 ml for the phases IIa and IIb and another at 1229 ml when merging phases II and III, both in the range 900–1500 ml and near the 1% level. (Fig 12). Nevertheless, only 25 vessels are attributed to the phase III and the ultimate results maybe be largely influenced by the data from phase II. Accordingly, the remainder of the demonstration will focus on the results pertaining to phases I and II (a-b), which will be designated as early and late vessels, respectively, for the sake of simplicity.

As a preliminary observation, the two *quanta* (148 and 1227 ml) do not exhibit a straightforward arithmetic relationship, as 1227 is not close to an integer multiple of 148. Instead, they present an unintuitive ratio of 1 to 8.3. This situation indicates that the early and late samples should be considered has following distinct metrological considerations, which support the second hypothesis of a general sample featuring several units.

Furthermore, the results concerning the capacity of the early and late vessels are quite clear. In both cases, the probability that they were obtained by chance is less than 5%, and even equalling 1% for the phase II (Fig 12). Nevertheless, as mentioned before, CQA can sometimes produce false positive results. Therefore, another step of analysis consists in testing the *quantum* as a structuring unit of the distribution of each sample.

### 5.3. Metrological systems and origins

The previous results suggest that there is a high probability that the vessels from the Le Moulin necropolis at Mailhac were produced according to a capacity standard. Additionally, it is likely that these standards evolved over time and that at least two different units organised them, one during the Late Bronze Age (phase I), the other during the Early Iron Age (phase II). The following section seeks to confirm such hypothesis and to elucidate the manner in which each of the samples is structured by the aforementioned metrological units, as well as the extent to which this occurs.

The following step aim to determine whether the *quanta* deduced from the CQA are also reflected in the general structure of the samples, as seen through the FDA. In order to confirm even a basic metrological construction, the sample must be composed of these structuring units and some of their integer multiples and intelligible fractions. In other words, the main clusters of values illustrated by the FDA must correspond to a metrological structure organised around the desired *quantum* to confirm the results of the CQA and propose hypothesis about the use of a specific metrological system.

**Fig 13:**
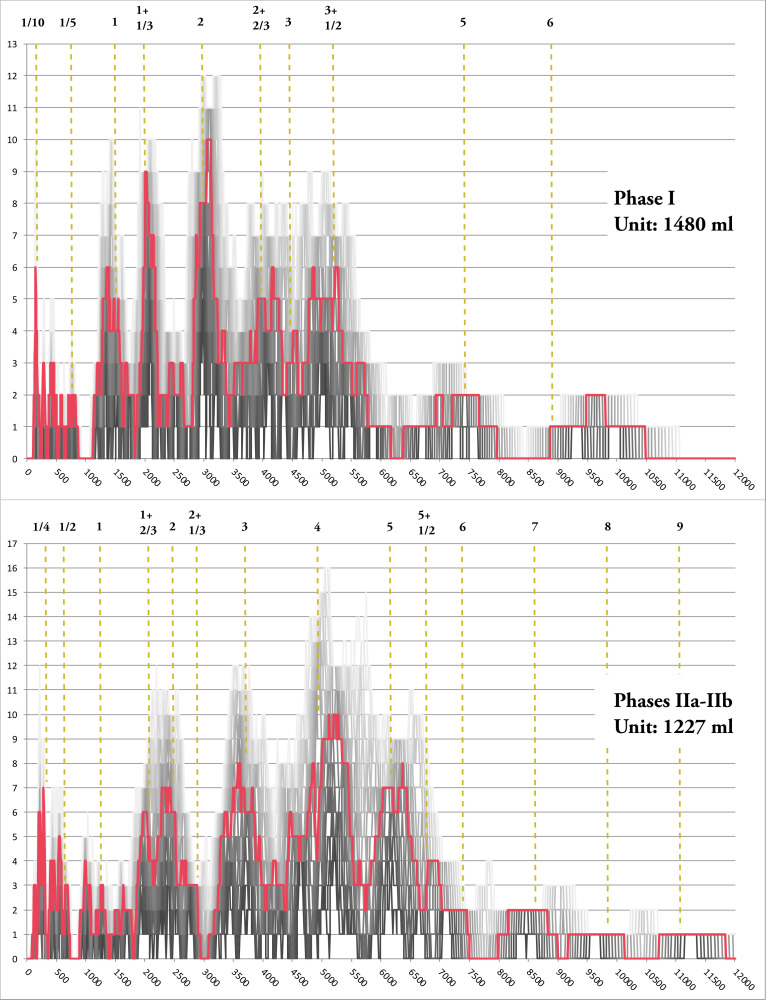
Frequency Distribution Analysis with tolerances and metrological system. FDA with tolerances from 1 to 10% (5% in red) and proposed metrological architecture of the samples of the Phase I (92 ind.) and Phase II (136 ind.).

For each phase, the FDA allow to validate the hypothetical unit resulting from the CQA. The distribution of values characteristic of the early phase can be explained by integral multiples of a 1480 ml unit (ten times the *quantum* of 148 ml): 1: 2: 3: 5: 6 (Fig 13). Most of the other values seem to correspond to half or third of this unit, and we observe values around 1 + 1/3, 2 + 2/3 and 3 + 1/2. The smaller values are harder to explain because of the poorer resolution in terms of capacity, but we can probably identify the fraction 1/10 that corresponds to the *quantum*. It is similarly conceivable that additional intermediate values may exist, albeit in a form that is challenging to delineate with precision, for example between 3 and 3 + 1/2. Similarly, capacities corresponding to about 6 units could correspond to a higher non-integer multiple (Fig 13; S3 Appendix).

In the same way, the 1227 ml *quantum* provides a very satisfactory explanation for the distribution of the late vessels dataset. Indeed, we can observe that most of the values are clustered around each integer multiple between 2 and 9. Looking at the submultiples, very frequent fractions appear, corresponding to half and quarter of the possible unit. It is likely that there are other submultiples. Unfortunately, as mentioned before, the smaller vessels are the least reliable for the study. Intermediate values of 1 + 2/3, 2 + 1/3 and 5 + 1/2 also appear.(S3 Appendix).

Despite a difference in the structuring unit, we can observe that the two samples share common features considering their distribution. Most of the elements are gathered within a similar interval, between 0 and 7000 ml. They also share a common logic, using thirds to increase the values of the unit and its double, and halves for the other multiples The existence of such values, especially in capacity metrology, is not unusual and can be explained by common standards, reduced or increased units (e.g., quantity for sale or taxation vs. quantity for purchase or distribution), or differences between dry and liquid quantities [44].

In Mailhac they are not so numerous as to obscure the reading of the results. Some capacity standards, especially those used in the Middle Ages, would be much more difficult to understand with our method because of functional and contextual differences for each different unit [44].

These different observations lead us to conclude that there was probably a metrological logic in the manufacture of vessels at Mailhac. This result is based on a series of converging evidence points: general distributions that do not follow the normal law, with concentrations of values around specific capacities; the inadequacy between these concentrations of values and morphological or typological criteria; CQA that return promising but distinct quanta for Phases I and II; confirmation of the viability of these quanta as structuring units of the samples from these phases; the similar construction of the samples from the two phases with a few fractions, numerous integer multiples and intermediate values made up of halves and thirds of units. All these criteria, taken together, lead us to propose the use of capacity metrology at Mailhac, from the Late Bronze Age onwards.

Concerning the genesis of such system, the question of the origin of Late Prehistoric metrological systems is not new, and a lot of scholars have already written on that topic, especially concerning weighing systems. One of the most frequently proposed explanation is that Bronze or Iron Age populations adopted metrological systems that had previously been used in Mediterranean regions. If such influences have been demonstrated, it is also evident that local dynamics played an important role.

An external origin for the metrological systems could therefore be a tempting hypothesis. Nevertheless, as previously stated, despite the proximity of the Mailhac sites to the Mediterranean and the fact that further developments will reinforce their connectivity with foreign merchants and Mediterranean populations, the sites remained relatively isolated during the Late Bronze Age period. The ceramic production is inscribed in a relatively small geographical influence extending from *Provence* to the *Empurdan* [19,45,46]. Similarly, the metallic objects are likely to have been produced locally. Only occasional imports from other European Late Prehistorical groups, especially the northern Alps, and potential influences or imports from the Italic horizon or the Eastern Mediterranean (two double-spring fibula and two arrows heads) [19]. Nevertheless, the aforementioned evidence for direct or indirect contacts is predominantly attributed to phase II and they cannot be considered as systemic due to the limited evidence. Therefore, there is no compelling reason to suggest that they adopted a foreign metrological system dedicated to vessel capacity, whilst vessels themselves do not move from one region to another.

However, it is generally impossible to validate or invalidate the hypothesis of the diffusion of a metrological unit from a comparative standpoint [4,6,33]. Firstly, capacity metrology is not a field of research that has been extensively investigated to date. Despite recent advances, there is not yet a sufficiently comprehensive comparative dataset for the periods in question in Western Europe and the Mediterranean. Secondly, the capacity units identified are typically employed with a relatively broad tolerance, which compromises the precision of comparison in the absence of other corroborating evidence for contacts. It has been demonstrated on numerous occasions that comparative metrology is subject to significant constraints. It is relatively straightforward to identify a unit of comparison that corresponds to a fraction or multiple of the unit under investigation through the application of elementary mathematical operations and varying tolerance thresholds. Consequently, the same protocol may result in disparate origins being attributed to the same metrological unit, rendering such an approach unscientific.

## 6. Discussion

The objective of this study was to ascertain whether evidence could be found for the utilisation of capacity measurement in Late Prehistoric Western Europe. In this regard, the sample from the *Nécropole du Moulin* at Mailhac yields particularly noteworthy findings.

As previously stated, the sample under examination is susceptible to a number of distortions. The vessels are not wheel-thrown and are sometimes asymmetrical, a factor not directly taken into account by the 3D calculation protocol. Furthermore, the accuracy expected by the potter or the users is unknown. The vessels buried with the deceased are also selected, which introduces a further potential source of bias as the sample is maybe not representative of all daily activities, in particular some underrepresentation of larger vessels. Moreover, the drawings made by archaeologists may not be entirely accurate. Potential scaling problems must also be considered. And the 3D protocol applied may present problems of precision. Finally, it should be noted that the metrological analyses themselves have limitations. However, our approach here has been to calculate the deviations induced by these approximations and to use analysis methods that enable us to avoid false positives and to detect evidence of metrological constructions, when they exist, even within small samples or with wide tolerance margins.

These results lead us to suggest that there was a real metrological desire to produce pots of a given capacity, based on specific units, which existed at Mailhac from the end of the Bronze Age.

The emergence of metrological thinking is often seen as a major symbolic and conceptual step in the human mind. Indeed, it is the evidence of the layering of abstract concepts – numbers, quantities, measurements, units – used to structure social and economic practices. It is not just a technical device, but an abstract way of ranking and sorting the world. Metrology, as quantitative, is first and foremost a social consensus based on the formulation of a concept of measurement and then the agreement on a unit of measurement as a quantifying tool [47]. However, metrological features do not only emerge as a consequence of an abstract and theoretical development such as the metric system. They can be the result of an empirical practice consisting in defining basic units in order to multiply or subdivide them to facilitate daily management or larger administrative tasks.

In this sense, metrology does not have to be perfectly accurate, as we are often led to believe. Accuracy is a social construct, and margins of approximation could have been wide, depending on the situation. For most tasks, a simple proportional system will suffice. One person needs a vase with twice the capacity of another because he needs it to hold food for two meals. Distribution processes easily generate this kind of technical need. If you know the capacity of a storage container, you can estimate how many people it can feed and how many days a family can live on what is stored inside. This can be compared to a chain of relationships that fit together like Matryoshka dolls.

In this sense, metrological practices can appear through two historical trajectories. The first is a bottom-up development, when people develop objects with increasingly homogeneous dimensions, whether as a consequence of production patterns or in response to everyday management issues such as production, storage or exchange. Another major top-bottom trajectory occurs when political entities promote or impose norms within production or exchange. Such a situation can result from various political, social as well as economic needs, such as the assertion of authority, the need to regulate distribution or trade processes, or the administrative facilitation of tax collection.

The Mailhac case developed in this article is a pilot study in an area of research where almost everything is still to be done. Consequently, the lack of comparative data greatly affects the conclusions that can be drawn in that sense. Nevertheless, it is possible to make a number of observations and discussions on the results, which must be considered as isolated and preliminary research.

As a first observation, the capacity metrology at Mailhac appears early, during the Late Bronze Age, at a time when we know very little about other measurement systems in the area and when the local population is not yet deeply involved in long-distance trade. They are also pottery vessels of local manufacture and local form. In this sense, there is nothing to suggest any kind of external influence in the production of the vessels studied. In such a situation, it seems logical to attribute a local origin to the metrological practices observed, if not out of a purely diffusionist attitude towards data. At this stage, the sample of vessels already shows a relatively high degree of uniformity and precision, which makes it possible to reconstruct a fairly complete metrological system based on a structuring unit of 1480 ml. This situation means that we cannot conclude on the modalities of appearance and development of this metrology. In order to achieve this goal, it will be necessary to make similar observations for earlier periods and neighbouring regions in order to replace this process in a dynamic way. Nevertheless, we can say that it probably means that people had already reached a high level of cognitive development in the abstraction and conceptualisation of measurement, as suggested by the high number of multiples represented, the use of fractions to obtain intermediate values, as well as the wide range of ceramic shapes and functions involved in the practice.

Another important insight comes from the evolution that can be observed between phases I and II. The various analyses show that the main characteristics of the practice do not change much: the range of capacities remains broadly similar, there are still different shapes involved without a correlation between shape and capacity, the system is still based on several multiples and some intermediate values. The only major change is in the unit structuring the metrological system, which has been reduced to 1227 ml. It can also be observed that this change must have occurred rather quickly, since the phase IIa already shows no sign of the previous unit (Table 1). This strong difference between the sample of phase I and IIa also leads to the rejection of the hypothesis of a slow reduction of the structuring unit. Such a situation should lead to a more nuanced sample for each phase and a subtler evolution between them. The data are therefore consistent with the introduction of a new unit replacing the previous one in a short period of time, at least on the archaeological scale of observation.

As mentioned above, there are few things that generally lead to the adoption of a new metrological unit: a strong change in practice, a political decision or an adaptation to external trade. Here, there seems to be no evidence of a change in pottery production that was innovative enough to influence a subsequent metrological consideration, nor that the vessels were involved in new trade networks, which leaves us with the hypothesis of political influence. However, the nature of such influence can vary greatly depending on the way people are governed, their main socio-economic structures and the way pottery production is embedded in this system. Our knowledge of such immaterial processes in the case of Mailhac is too weak to fully support the hypothesis and explore it further, despite the fact that the transition between Phases I and II of the necropolis also corresponds to the first abandonment of *Le Cayla* dwelling. We will simply note that if we attribute a political origin to the metrological shift between Phase I and Phase II, it has strong consequences for the role played by capacity metrology for the populations concerned and their embedding in societies. Such a statement differs greatly from the traditional image of these populations, to which we attribute weak political structures and a relatively passive role in socio-economic developments in the Western Mediterranean, in particular regarding metrology.

## 7. Conclusion

In this paper we have shown that at Mailhac, there are strong evidence to support the hypothesis that at least part of the pottery production was made according to capacity metrology as early as the Late Bronze Age, several centuries before the Roman expansion and the traditional time when we consider that capacity standards were used in Southern France. Obviously, because of the biases introduced by the metrological study of pottery vessels, we can hardly assess the degree of precision that was actually expected by pottery makers and users.

Metrology is an abstract process that allows individuals and societies to effectively structure their world [47], and measurements are cognitive constructions that allow us to interact with the world in a particular way. But their existence and use has a feedback effect on how people think about the world.

With this paper we have shown that the ability of Late Bronze Age and Early Iron Age societies from Western Europe to manipulate abstract concepts in order to interact with their natural environment and to create new institutions should not be underestimated. We already knew that they manipulated weights and scales since at least the Middle Bronze Age, a phenomenon often associated with long-distance trade. However, this study shows that by the Late Bronze Age, the mastery of cognitive tools such as metrological systems had already deeply conditioned the social practices and daily activities of the community inhabiting Mailhac. More than the production of standardised pots, these results suggest a daily interaction between people and measurements for storing, cooking, drinking and consuming food.

Such a result forces us to completely rethink our representations of Bronze and Early Iron Age societies in Western Europe. At least three centuries before the founding of Massalia, and several centuries before the Roman expansion into southern France, some people were already using various metrological systems, probably with deep ramifications in their daily lives. As a result, they already had the cognitive tools to carry out the complex abstract processes that are a prerequisite for administration or economic calculation. If such a situation has generally been denied, it is because there is no other evidence of the social complexity associated with metrological practices. The absence of such evidence is undoubtedly a sign that other incentives conditioned the adoption and use of measurement. This article represents a pilot study that does not yet allow us to place the observations and hypotheses put forward in a broader chrono-cultural framework. It is therefore not yet possible to determine whether the situation at Mailhac is the exception or the rule in the region. These results do, however, show just how interesting it is to take up the subject of capacity metrology, even for early periods of European Late Prehistory and corpuses of handmade vessels. This would be the basis for new research on the extent and role of metrology in ancient European societies.

## Supporting information

S1 Appendix Inventory of the corpus of vessels studied and analysed. (XLSX)

S2 Appendix Results of the asymmetry simulations. (XLSX)

S3 Appendix Proposition of metrological system used at Mailhac for the Phases I and II. (XLSX)
